# Changes in Serum Electrolytes, ECG, and Baroreflex Sensitivity during Combined Pituitary Stimulation Test

**DOI:** 10.1155/2018/8692078

**Published:** 2018-05-09

**Authors:** Sungsu Kim, Choong Hwan Kwak, Jaehoon Jung, Jong Ha Baek, Jung Hwa Jung, Ki-Jong Park, Kyongyoung Kim, Soo Kyoung Kim, Dawon Kang, Jong Ryeal Hahm

**Affiliations:** ^1^Division of Endocrinology and Metabolism, Department of Internal Medicine, ISAM Hospital, Busan, Republic of Korea; ^2^Division of Cardiology, Department of Internal Medicine, Gyeongsang National University Changwon Hospital, Changwon, Republic of Korea; ^3^College of Medicine and Institute of Health Sciences, Gyeongsang National University, Jinju, Republic of Korea; ^4^Division of Endocrinology and Metabolism, Department of Internal Medicine, Gyeongsang National University Changwon Hospital, Changwon, Republic of Korea; ^5^Division of Endocrinology and Metabolism, Department of Internal Medicine, Gyeongsang National University Hospital, Jinju, Republic of Korea; ^6^Department of Neurology, Gyeongsang National University Changwon Hospital, Changwon, Republic of Korea; ^7^Department of Physiology, College of Medicine and Institute of Health Sciences, Gyeongsang National University, Jinju, Republic of Korea

## Abstract

The mechanisms by which hypoglycemia increases cardiovascular mortality remain unclear. The aim of the study is to investigate changes in serum electrolytes, norepinephrine concentrations, electrocardiography, and baroreflex sensitivity (BRS) and associations between corrected QT (QTc) intervals and the changes in serum electrolytes during combined pituitary stimulation test (CPST). We recruited the subjects who were admitted to the Gyeongsang National University Hospital to undergo CPST between September 2013 and December 2014. Participants were 12 patients suspected of having hypopituitarism. Among 12 patients, cardiac arrhythmia in two patients occurred during hypoglycemia. There were significant differences in serum levels of potassium (*P* < 0.001), sodium (*P* = 0.003), chloride (*P* = 0.002), and calcium (*P* = 0.017) at baseline, hypoglycemia, and 30 and 120 minutes after hypoglycemia. Also, there was a significant increase in heart rate (*P* = 0.004), corrected QT (QTc) interval (*P* = 0.008), QRS duration (*P* = 0.021), and BRS (*P* = 0.005) at hypoglycemia, compared to other time points during CPST. There was a positive association between QTc intervals and serum sodium levels (*P* < 0.001) in 10 patients who did not develop arrhythmia during CPST. This study showed that there were significant changes in serum levels of potassium, sodium, chloride, and calcium, as well as heart rate, QTc interval, QRSd, and BRS during CPST. It was revealed that QTc intervals had a significant association with concentrations of sodium.

## 1. Introduction

Large randomized clinical trials (RCT) have shown that intensive glycemic control prevents the development and progression of diabetic microvascular complications in patients with diabetes mellitus (DM), compared with conventional treatment [1–3]. However, it is unclear whether this intervention exhibits similar effects on cardiovascular events in patients with type 2 diabetes and prolonged disease duration [3–5]. The findings of the Action to Control Cardiovascular Risk in Diabetes (ACCORD) trial in 2008 showed increased cardiovascular or all-cause mortality in the intensive therapy group compared with standard therapy [4], which led to early termination of the ACCORD trial.

Meta-analyses of RCTs indicated that severe hypoglycemia frequently occurred in the intensive glycemic control group [6, 7]. A causal link between insulin-induced hypoglycemia and death was demonstrated by a case report that showed the presence of hypoglycemia, which was documented by continuous glucose monitoring data, at the time of the death of a young patient with type 1 diabetes [8]. The results from the Normoglycemia in Intensive Care Evaluation-Survival Using Glucose Algorithm Regulation (NICE-SUGAR) [9] and Control of Hyperglycemia in Pediatric Intensive Care (ChiP) [10] indicated an association between intensive glycemic control with insulin and increased mortality. Furthermore, studies have suggested that iatrogenic hypoglycemia, caused by administration of insulin or of an insulin secretagogue, was associated with the death of patients with diabetes [11, 12]. Although the mechanisms by which hypoglycemia increases cardiovascular mortality remain unclear, arrhythmic deaths were reported as a direct cause of the mortality in the NICE-SUGAR trial [9]. A report from the Outcome Reduction with Initial Glargine Intervention (ORIGIN) trial demonstrated that severe hypoglycemia was associated with a 77% greater risk of arrhythmic death [13]. A clinical study using combined Holter electrocardiography (ECG) and continuous glucose monitoring showed that cardiac arrhythmias, which were related to corrected QT (QTc) interval prolongation, occurred during moderate hypoglycemic periods in patients with type 1 diabetes [14]. QTc interval prolongation in patients with long QT syndrome (LQTS) increased the risk of ventricular arrhythmia that might lead to sudden death [15].

The combined pituitary stimulation test (CPST) is a medical diagnostic procedure for simultaneously assessing hypofunction of anterior pituitary hormones. In fact, it is difficult to demonstrate how fatal cardiac arrhythmias are mediated during the insulin-induced severe hypoglycemic period. As insulin was injected as a bolus in the patient's vein to cause hypoglycemia for CPST, this gave us an opportunity to study the underlying link between the iatrogenic hypoglycemia and cardiac arrhythmia. The aim of this study was to investigate the changes in serum electrolytes, norepinephrine concentrations, ECG, and baroreflex sensitivity (BRS) and the associations between QTc intervals and the changes in serum electrolyte during CPST.

## 2. Methods and Materials

### 2.1. Study Subjects

Subjects admitted to Gyeongsang National University Hospital to undergo CPST were enrolled for this study. The study protocol was approved by the Ethical Committees at Gyeongsang National University Hospital (IRB number 2012-10-006). All participants provided written informed consent.

### 2.2. Combined Pituitary Stimulation Test

All subjects did not have any intake of food, except for water, for at least eight hours and remained conscious in a supine position throughout CPST. An intravenous catheter was inserted into a vein in the back of hand/arm and infused with 0.9% normal saline to keep the intravenous line open. Two releasing hormones and insulin were injected intravenously from separate syringes in the following order and doses: thyrotropin-releasing hormone (TRH; 200 *μ*g), gonadotropin-releasing hormone (GnRH; 50 *μ*g), and insulin (0.10 U/kg). More insulin was given intravenously in a dose of 0.05 U/kg if a patient did not develop hypoglycemia after injection of that dose of insulin. All CPSTs were performed under the supervision of a doctor, who examined the subjects for development of symptoms of hypoglycemia such as shaking, sweating, and drowsiness. Insulin-induced hypoglycemia was defined as a blood glucose level below 40 mg/dL or having hypoglycemic symptoms and a blood glucose level below 50 mg/dL. Twenty percent dextrose solution was used to restore the blood glucose level to normal after the subject developed hypoglycemia.

### 2.3. Measurements

Blood samples and ECG data were collected at baseline, hypoglycemia, and 30 and 120 minutes after hypoglycemia. Serum glucose and electrolytes levels, including potassium, sodium, chloride, and calcium, were measured using a Cobas 8000 (Roche, Mannheim, Germany). Blood samples for norepinephrine analysis were collected in chilled ethylenediaminetetraacetic acid (EDTA) tubes at baseline, hypoglycemia, and 30 minutes after hypoglycemia, and serum norepinephrine level was analyzed by radioimmunoassay.

Heart rate was calculated from the ECGs taken at consecutive times. The cardiologist, blinded to data such as medical information and time of ECG measurements, measured the PR interval, QRS duration (QRSd), and QT interval. The QT interval was measured by a standard method. Briefly, the onset of the Q wave from average beats derived from leads I, II, and V5 was marked as the first positive deflection from the isoelectric line >10 microvolts, and the end of the T wave was determined using the tangent method. QTc interval was calculated using Bazett's formula: (QT interval)/(square root of R-R interval). For the assessment of autonomic function, the measurement of BRS was performed sequentially during CPST by using a Finometer (Finapres Medical Systems BV, Amsterdam, Netherlands).

### 2.4. Statistics

Data were expressed as the mean ± standard deviation (SD) and frequency (%). The Shapiro-Wilk test for normality was performed to determine if the data were normally distributed. To perform a comparison of repeated measurements during CPST, one of repeated measures analysis of variance (RM-ANOVA), linear mixed model (LMM), or Friedman test was selected depending on distribution and availability of measurement. If there were significant differences in measurements over time, Tukey's post hoc analysis was conducted to determine differences between consecutive time points. A linear mixed model with random intercepts or slopes for the by-subject was constructed to examine the relationship between QTc interval and serum electrolyte. The proper model was selected by the Akaike information criterion (AIC) or log likelihood value. Visual inspection of residual and quantile-quantile (Q-Q) plots was carried out to examine any apparent deviations from homoscedasticity or normality of residuals. Likelihood ratio tests were checked as a means to attain *P* values for fixed effects. For all the analyses, *P* values ≤ 0.05 were considered statistically significant. All statistical analyses were performed by R statistics version 3.2 (R Foundation for Statistical Computing, https://www.R-project.Org) and lme4 (Bates, Maechler, Bolker and Worlker, 2015).

## 3. Results

### 3.1. Study Participants

Between September 2013 and December 2014, a total of 12 patients were enrolled into the study. The mean (±SD) age of patients was 49 ± 12.8 years, and the percentage of male subjects was 33%. Of the 12 patients, six had pituitary adenoma; four underwent transsphenoidal adenoidectomy; and two patients were diagnosed as having a prolactinoma and had been given bromocriptine. Six patients had no medications before admission for CPST, and two patients had received antihypertensive medication, one of whom had a medical history of diabetes. The clinical characteristics of enrolled subjects are presented in Table 1.

### 3.2. Changes in Serum Electrolyte and Glucose Levels and Norepinephrine Concentrations

**Figure 1:**
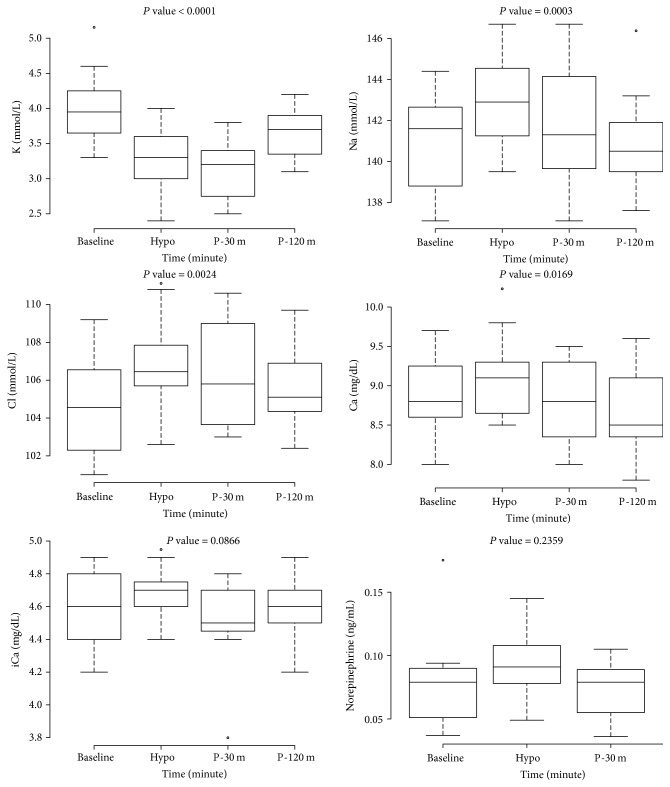
Changes in serum electrolytes and norepinephrine during combined pituitary stimulation test. *P* value was attained by repeated measures analysis of variance or linear mixed regression. Hypo, at hypoglycemia; P-30 m, at 30 minutes after hypoglycemia; P-120 m, at 120 minutes after hypoglycemia.

There were significant differences in serum levels of potassium (*P* < 0.001), sodium (*P* = 0.003), chloride (*P* = 0.002), calcium (*P* = 0.017), and glucose (*P* < 0.001) at baseline, hypoglycemia, and 30 and 120 minutes after hypoglycemia (Figure 1 and Supplemental Figure 1). Of the four time points, the median serum potassium level at 30 minutes after hypoglycemia was lowest, and the median serum sodium, chloride, and calcium levels at hypoglycemia were highest. Post hoc analysis showed statistically significant changes in serum potassium levels between baseline and hypoglycemia (*P* < 0.001), between baseline and 30 minutes after hypoglycemia (*P* < 0.001), and between 30 and 120 minutes after hypoglycemia (*P* = 0.019); in serum sodium levels between baseline and hypoglycemia (*P* = 0.001) and between hypoglycemia and 120 minutes after hypoglycemia (*P* = 0.032); in serum chloride levels between baseline and hypoglycemia (*P* = 0.016); in serum calcium levels between hypoglycemia and 120 minutes after hypoglycemia (*P* = 0.046); in serum glucose levels between hypoglycemia and the other time points (Supplemental Figure 1). There were no significant differences in serum ionized calcium (iCa) and norepinephrine concentrations, which were measured at three time points, in seven patients (Figure 1).

### 3.3. Changes in ECG and BRS

**Figure 2:**
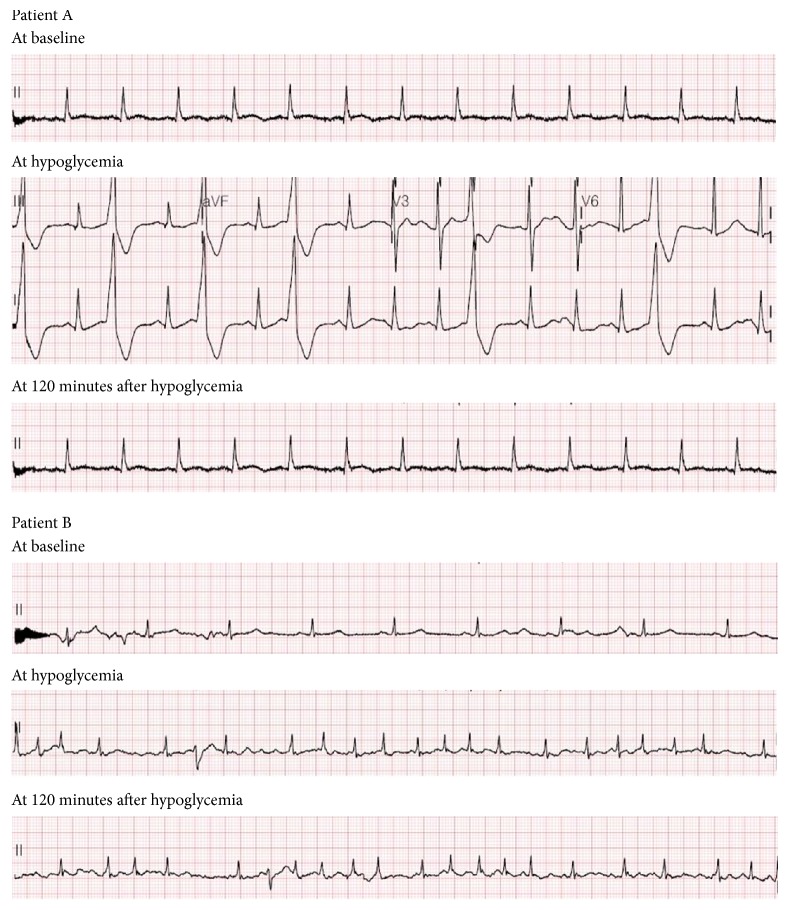
Development of arrhythmia at hypoglycemia in two patients. Patient A and Patient B are subjects who developed arrhythmia during combined pituitary stimulation test.

**Figure 3:**
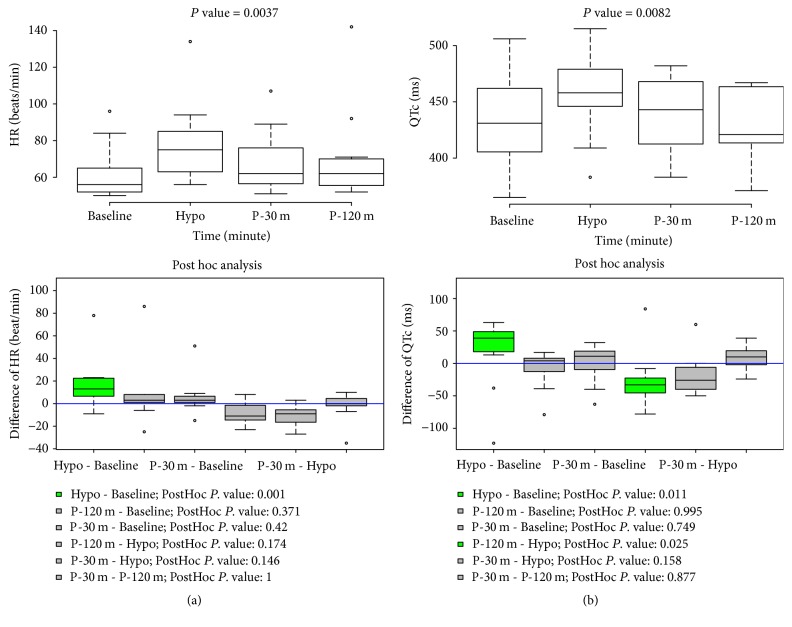
Changes in heart rate and QTc during combined pituitary stimulation test. *P* value was attained by Friedman test. Hypo, at hypoglycemia; P-30 m, at 30 minutes after hypoglycemia; P-120 m, at 120 minutes after hypoglycemia.

**Figure 4:**
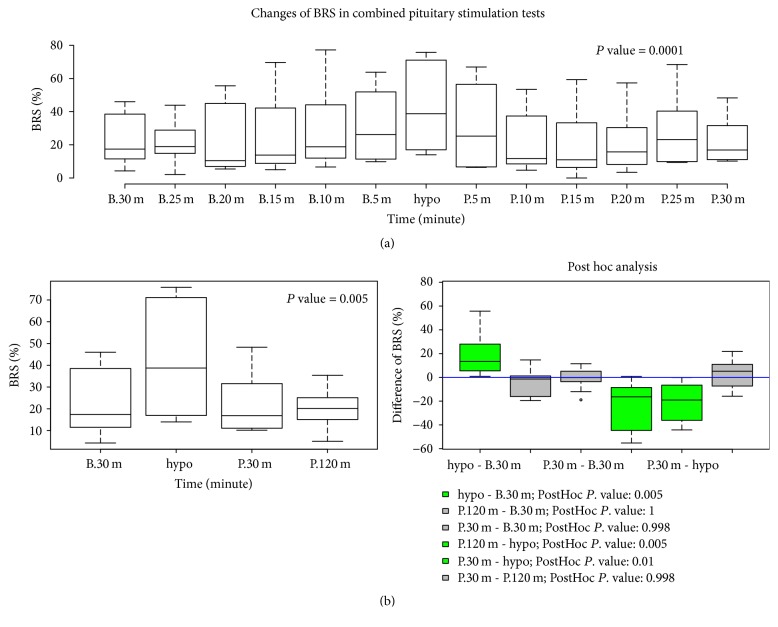
Changes in baroreflex sensitivity during combined pituitary stimulation test. *P* value was calculated by Friedman test. B.30 m, 30 minutes before hypoglycemia; Hypo, hypoglycemia; P.30 m, 30 minutes after hypoglycemia; P.120 m, 120 minutes after hypoglycemia.

Cardiac arrhythmia in two patients occurred at hypoglycemia. One with type 2 diabetes and hypertension developed ventricular premature beats at hypoglycemia and recovered at 120 minutes after hypoglycemia. The other with past medical history of diffuse large B-cell lymphoma developed atrial fibrillation (AF) at hypoglycemia but did not recover from AF until 120 minutes after hypoglycemia (Figure 2). T wave changes were observed in most participating patients during CPST: T wave in lead II was reduced in amplitude at hypoglycemia but partially recovered from the flat T wave at two hours after hypoglycemia (Supplemental Figure 2). Heart rate, QTc, and QRSd were significantly changed during CPST. There were significant increases in these measurements at hypoglycemia compared with those at the other time points. Post hoc analysis indicated that there were significant changes in heart rate between baseline and hypoglycemia (*P* = 0.002); in QTc interval between baseline and hypoglycemia (*P* = 0.011) and between hypoglycemia and 120 minutes after hypoglycemia (*P* = 0.023); and in QRSd between hypoglycemia and 120 minutes after hypoglycemia (*P* = 0.045) (Figure 3 and Supplemental Figure 3). PR and QT intervals had no significant differences at these times (Supplemental Figure 3). There were significant differences in BRS for one hour before and after hypoglycemia. The median BRS at hypoglycemia was the highest for 1 hour. During four time points, that is, 30 minutes before hypoglycemia, during hypoglycemia, and 30 and 120 minutes after hypoglycemia, there were statistically significant changes in BRS. Post hoc analysis revealed that there was a significant increase in BRS at hypoglycemia compared with those seen at other time points (Figure 4).

### 3.4. Associations between QTc Intervals and Changes in Serum Electrolyte Levels

**Figure 5:**
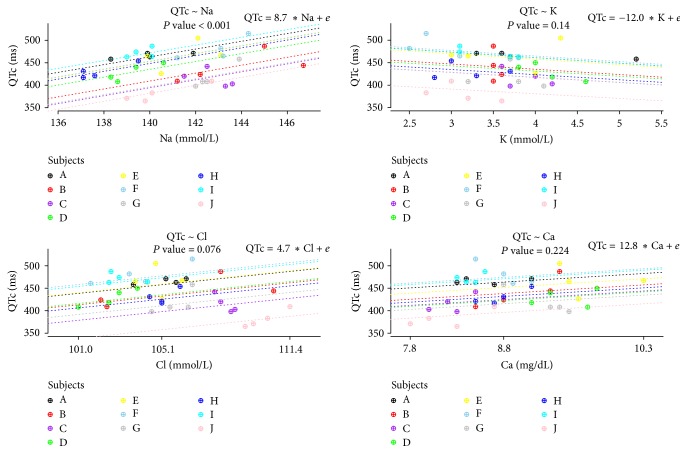
The associations between QTc intervals and the changes of serum electrolytes levels. *P* value was attained by likelihood ratio tests. The letters A–J indicate individual subjects, and symbols in the plots, which have the same color matched with the letters, denote repeated measurements within an individual. Dotted lines indicate linear regression equations from a linear mixed model with random intercepts or slopes for the by-subject.

Linear associations between QTc intervals and changes in serum electrolyte levels were examined in 10 patients who did not develop arrhythmia at hypoglycemia. As shown in Figure 5, a strong positive association between QTc intervals and serum sodium levels was observed. With each mmol/increase in serum sodium, the prolongation of QTc interval was about 8.7 milliseconds (*P* < 0.001). But there were no significant associations between QTc intervals and serum levels of potassium, chloride, and calcium (Figure 5). We investigated associations between serum glucose levels and the parameters in addition. Serum sodium levels (*P* = 0.010), QTc intervals (*P* = 0.032), and BRS (*P* = 0.024) had significant associations with the serum glucose levels (Supplemental Table 1).

## 4. Discussion

The present study showed that patients who were suspected of having hypopituitarism had significant differences in serum levels of potassium, sodium, chloride, and calcium, as well as heart rate, QRSd, QTc, and BRS at hypoglycemia, compared to other time points during CPST. Also, there was a positive association between QTc intervals and serum sodium levels, which were measured at baseline, hypoglycemia, and 30 and 120 minutes after hypoglycemia. A decrease in serum potassium occurred during hypoglycemia, and a steady decrease was observed until 30 minutes after hypoglycemia compared with serum potassium levels at baseline. Multiple studies suggest that the effects of insulin-induced hypoglycemia on serum potassium may be the main mechanisms of direct or indirect activation of membrane-bound Na^+^/K^+^ ATPase. In an experiment using frog skeletal muscle, it was reported that insulin directly stimulates membrane-bound Na^+^/K^+^ ATPase and increases intracellular potassium uptake [16]. In healthy men, DeFronzo et al. [17] demonstrated an insulin dose-dependent decline in serum potassium by using the hyperinsulinemic euglycemic insulin clamp. In rat soleus muscle [18] and normal subjects [19], hypokalemia caused by epinephrine infusion activated Na^+^/K^+^ ATPase through the stimulation of *β*_2_ adrenoceptors. These findings suggested that counterregulatory secretion of epinephrine, which occurs during severe hypoglycemia, could be an indirect cause for the decline in serum potassium. The changes in serum potassium in this study are consistent with the results of a previous study by Petersen et al. [20]. They observed that the decrease in serum potassium during insulin-induced hypoglycemia had a two-stage process with an initial fall and an additional decline, which was prevented in subjects given propranolol [20]. This revealed that the initial fall in serum potassium was associated with the intravenous insulin-injection and that the further decline in serum potassium had an association with an increase in serum catecholamines. An important role of aldosterone in plasma potassium homeostasis could be another possible mechanism for the slow recovery of serum potassium. In hypoglycemia, the activation of the renin-angiotensin system caused an increase in plasma aldosterone [21] which in turn increased renal potassium excretion [22].

Although insulin-induced hypokalemia is well known, few studies have explored the changes in serum sodium, chloride, and calcium in response to hypoglycemia. The changes in these electrolytes followed a similar pattern in this study; the plasma concentrations in the electrolytes at hypoglycemia increased and reverted rapidly to baseline levels at 30 minutes after hypoglycemia. Caduff et al. [23] performed a study that investigated the changes in major ions, namely, sodium, chloride, and calcium, in subjects with type 1 diabetes, using the glucose clamp technique. They reported that changes in these ion concentrations reflected the blood glucose levels. This is in agreement with the results of our study. The increase in serum sodium concentrations at hypoglycemia might be induced by insulin-activated membrane-bound Na^+^/K^+^ ATPase and hypoglycemia-downregulated sodium glucose linked transporter 1 (SGLT1) in several tissues; in turn, it leads to an outflux of intracellular calcium through the Na^+^ gradient. This can explain the elevation of levels of the two serum electrolytes during hypoglycemia. But there were no significant differences in ionized calcium concentrations so that there might be other mechanisms that could explain the difference in the results between serum total and ionized calcium levels. Body et al. [24] reported that smaller increments in both total and ionized calcium in healthy men who developed insulin-induced hypoglycemia resulted from endogenous epinephrine secretion caused by hypoglycemic stress. Our study included the subjects with hypopituitarism who had inadequate counterregulatory hormonal responses and thus might result in more profound systemic changes in insulin-induced hypoglycemia. These differences of systemic responses in stress hormones, induced hypoglycemia, among subjects in the two studies might result in the inconsistency in the results of changes in ionized calcium levels, and it would be clarified in the future. The return to baseline concentrations of these ions at 30 minutes after hypoglycemia without an additional increase might be associated with a net flow of water from cells to blood, which could be caused by the administration of 20% dextrose solution.

In the case of effects of insulin-induced hypoglycemia on ECG, this study showed that there was the development of arrhythmia in two patients and the increase in heart rate, QRSd, and QTc interval at hypoglycemia compared with the other time points during CPST. These changes in ECG have been reported by multiple studies. Insulin-induced hypokalemia and the secretion of adrenaline in response to insulin-induced hypoglycemia have been shown to be a principal cause of these alterations in ECG. Hypokalemia increases resting membrane potential and the duration of the action potential and refractory period in cardiac myocytes [25]. Lloyd-Mostyn and Oram [26] investigated the cardiovascular effects of hypoglycemia in healthy men with the administration of *β*-blockers. They showed that propranolol prevented an increase in heart rate and the development of arrhythmia during hypoglycemia. In a large clinical trial, Mellbin et al. [13] revealed that severe hypoglycemia was associated with a greater risk for arrhythmic death in patients with type 2 diabetes, prediabetes, or a prior cardiovascular event.

The baroreceptor reflex is a key mechanism to maintain blood pressure at constant levels. BRS is a measurement to quantify how much control the baroreflex has on heart rate. It has been shown that there was a significant increase in BRS at hypoglycemia compared with other time points. A rise in blood pressure during hypoglycemia was observed in patients with diabetes using a continuous glucose monitoring system and simultaneous ambulatory blood pressure measurement [27]. This hypoglycemic-induced blood pressure elevation could cause an increase in parasympathetic tone and a decrease in sympathetic tone. Decreased BRS as an early mark of cardiovascular autonomic neuropathy (CAN) was found in patients with diabetes [28]. Okada et al. [29] showed that BRS could predict cardiovascular events in patient with type 2 diabetes. In our study, the result of BRS suggests that an increase in BRS at hypoglycemia might prevent the development of tachycardia and arrhythmia.

The QTc interval represents the summation of the action potential of ventricular myocytes that have specialized channels made of protein complexes across their membrane. Mutations of the genes that encode these protein channels lead to congenital long QT syndrome (LQTS) [30]. It is well known that electrolyte disturbances cause acquired LQTS. Hypokalemia that may prolong the cardiac repolarization by reducing the delayed and inward rectifier potassium current [31] is the most common cause of acquired LQTS [32]. Mok et al. reported a case in which acquired LQTS was associated with hyponatremia [33]. However, in this study, QTc intervals had a more significant association with concentrations of serum sodium than those of serum potassium during CPST. It had been reported that, contrary to the widespread impression, the QTc interval prolongation was not by hypokalemia but by hypernatremia, and the apparent prolongation of the QTc interval in many cases with hypokalemia resulted from the U wave being superimposed on the descending limb of the T wave [34, 35]. We also found that the QTc interval was significantly associated with sodium increments but not potassium. On the basis of these findings, we presumed that the elevated serum Na+ levels increased during CPST, in our study, could be likely to directly prolong the QTc interval. Surawicz and Lepeschkin [34] had also shown that patients with hypokalemia and hypocalcemia developed increased QTc interval. There were significant increases in serum calcium levels at hypoglycemia in our study. Therefore, this study suggests that the concentration of sodium is a good indicator of QTc elongation during hypoglycemia.

There are limitations in our study. First, the sample size for this study is too small to reveal the association between QTc intervals and serum potassium levels and the changes in serum norepinephrine level which have been reported in studies [36, 37] investigating norepinephrine kinetics in response to insulin-induced hypoglycemia. Second, as the subjects suspected of having hypopituitarism were enrolled, the results of this study cannot be generalized and applied to patients with diabetes directly. We could not find out effects of other hormones including TRH, TSH, GnRH, and GH on QTc intervals because of these limitations. Altun et al. [38] showed serum TSH levels had positive correlations with QTc intervals during TRH test in 20 euthyroid healthy subjects. In spite of the limitations, we documented the development of arrhythmia in two subjects and showed that there were significant changes in serum levels of potassium, sodium, chloride, and calcium, as well as heart rate, QTc interval, QRSd, and BRS during CPST. It was revealed that QTc intervals had a significant association with concentrations of sodium. This study indicated that clinicians should check the serum sodium levels in patients during CPST to avoid ventricular arrhythmias. However, further researches that can demonstrate the exact role of insulin-induced electrolyte changes in prolonging QTc intervals are needed.

## Figures and Tables

**Table 1:** Clinical characteristics of the enrolled subjects.

Pt	Age	Sex	*H* (cm)	*W* (kg)	FBS (mg/dL)	HbA1c (%)	Underlying disease and history of operation	Medications
1	58	M	162.0	61.0	108	5.7	Pituitary adenoma	-

2	43	F	150.0	45.0	86	NA	Pituitary apoplexy	-

3	65	M	163.8	72.5	112	5.6	Pituitary adenoma, Diffuse large B-cell lymphoma, s/p TSA	Prednisolone

4	52	F	161.7	65.9	108	6.4	Prolactinoma, HTN	ARB, bromocriptine

5	42	F	161.8	64.6	83	5.5	Pituitary adenoma	-

6	22	F	161.9	50.6	100	NA	Hypogonadotropic hypogonadism	-

7	60	M	170.2	71.5	129	5.7	Pituitary adenoma	-

8	63	F	155.3	79.3	105	5.8	Empty sella syndrome, PTC, s/p total thyroidectomy	Cal.vit. D, LT4

9	43	F	162.2	68.7	99	NA	Prolactinoma	Bromocriptine

10	55	M	161.0	72.5	82	6.0	Pituitary adenoma, s/p TSA	Prednisolone, LT4, cal.vit. D, testosterone

11	33	F	157.0	44.0	88	NA	Pituitary adenoma, s/p TSA	-

12	52	F	160.5	72.3	121	6.4	Acromegaly, DM, HTN, s/p TSA,	Losartan, thiazide, metformin, sitagliptin

total 12	49 ± 12.8	M/F (33%/67%)	160.6 ± 4.9	63.9 ± 11.6	101.7 ± 15	5.9 ± 0.3	Pituitary adenoma: 6, s/p TSA: 4, Prolactinoma: 2, DM: 1, HTN: 2,	

Values are expressed as mean ± standard deviation or number (%). Pt, patients; *H*, height; *W*, weight; FBS, fasting blood sugar; HbA1c, glycated hemoglobin; NA, Not available; Cal.vit.D, calcium vitamin D; DM, diabetes mellitus; HTN, hypertension; LT4, levothyroxine; PTC, papillary thyroid carcinoma; s/p, status post; TSA, transsphenoidal adenoidectomy.

## Supplementary Materials

Supplementary Materials Supplemental Table 1: the associations between serum glucose levels and parameters. Supplemental Figure 1: changes in serum glucose levels during combined pituitary stimulation test. Supplemental Figure 2: T wave changes in lead II during combined pituitary stimulation test. Supplemental Figure 3: changes in QRS duration, PR interval, and QT interval during combined pituitary stimulation test.
