# Supplementary material for: Changes in Serum Electrolytes, ECG, and Baroreflex Sensitivity during Combined Pituitary Stimulation Test
Source: Biomed Res Int. 2018 May 9;2018:8692078. doi: 10.1155/2018/8692078 (PMC6076964; doi:10.1155/2018/8692078)
Supplement: Supplementary Materials — Supplemental Table 1: the associations between serum glucose levels and parameters. Supplemental Figure 1: changes in serum glucose levels during combined pituitary stimulation test. Supplemental Figure 2: T wave changes in lead II during combined pituitary stimulation test. Supplemental Figure 3: changes in QRS duration, PR interval, and QT interval during combined pituitary stimulation test. [file 8692078.f1.pdf]

Supplemental Table. 1. The associations between serum glucose levels and parameters.

Parameters	Fixed effects	95% CI	P value
Na (mmol/L)	-7.99	-14.0 to -2.0	0.010
K (mmol/L)	-8.36	-34.8 to 18.1	0.527
Cl (mmol/L)	-0.18	-5.7 to 5.3	0.948
Ca (mg/dL)	-22.27	-45.9 to 4.1	0.098
iCa (mg/dL)	-49.61	-108.9 to 11.6	0.111
QTc (ms)	-0.42	-0.8 to -0.1	0.032
HR (beats/min)	-0.97	-2.0 to 0.1	0.064
BRS (%)	-1.08	-1.9 to -0.3	0.024

P-value and fixed effects were attained by likelihood ratio tests and linear mixed model. CI, confidence interval; Na, serum sodium levels; K, serum potassium levels; Cl, serum chloride levels; Ca, serum calcium levels; iCa; ionized serum calcium levels; QTc, corrected QT intervals; HR, heart rate; BRS, baroreflex sensitivity.

Supplemental Fig. 1. Changes in serum glucose levels during combined pituitary stimulation test.

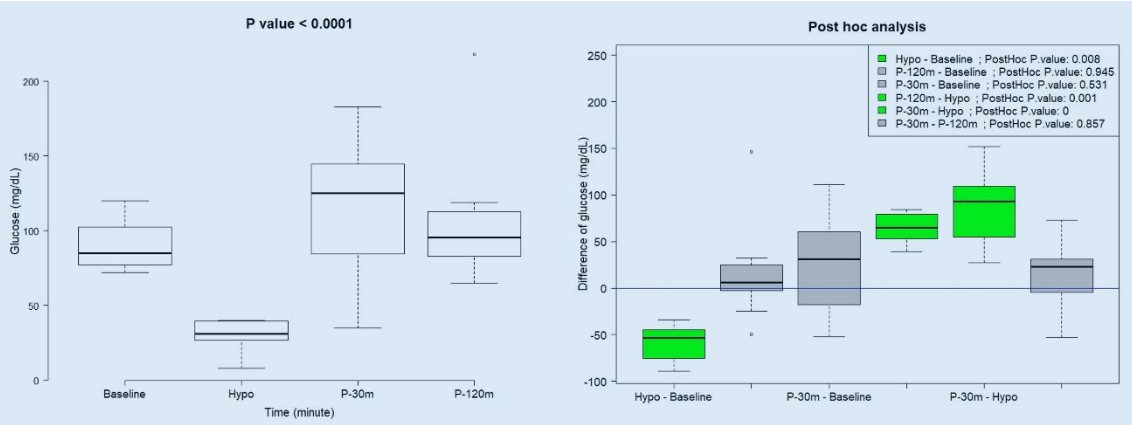

P value was attained by Friedman test. Hypo, at hypoglycemia; P-30m, at 30 minutes after hypoglycemia; P-120m, at 120 minutes after hypoglycemia.

Supplemental Fig. 2. T wave changes in lead II during combined pituitary stimulation test.

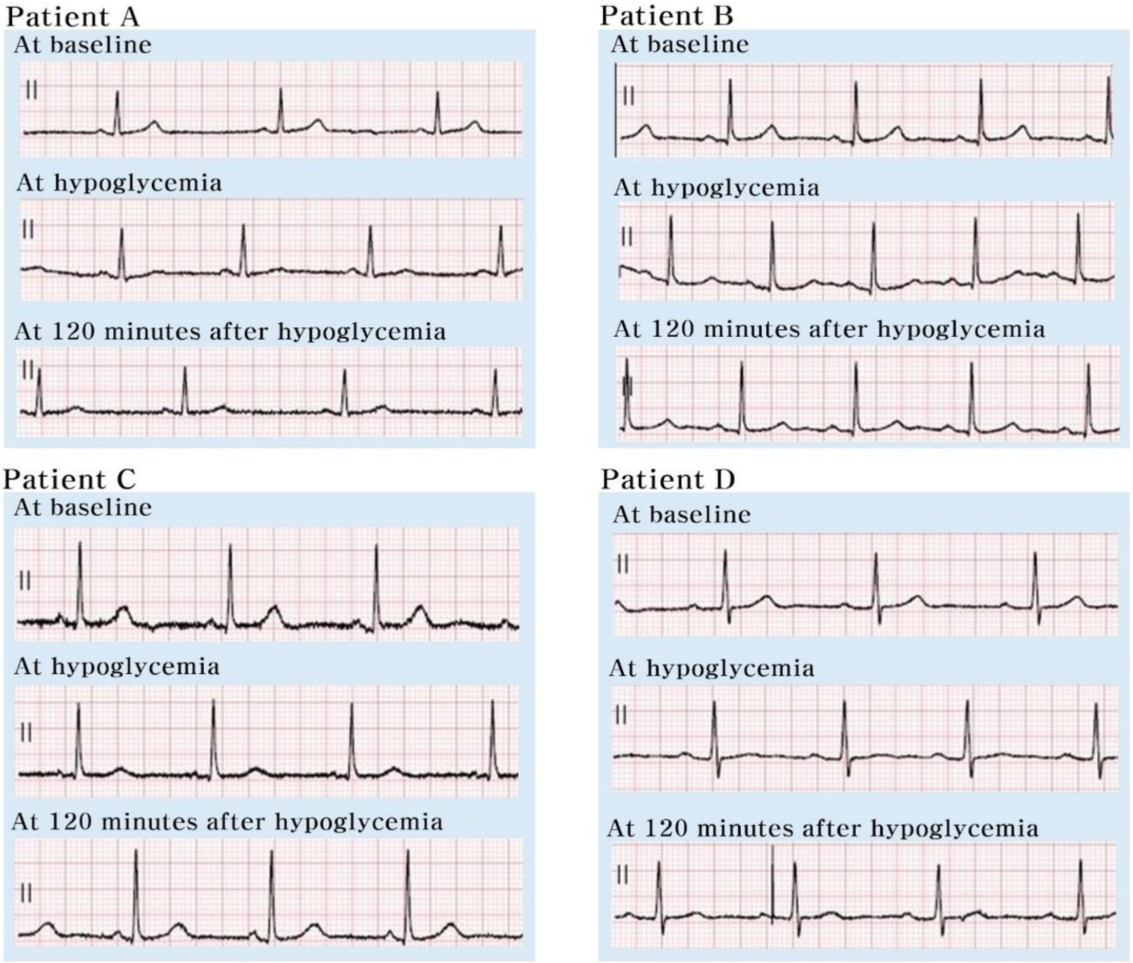

Patient A, B, C, and D indicate an individual enrolled subject

Supplemental Fig. 3. Changes in QRS duration, PR interval, and QT interval during combined pituitary stimulation test.

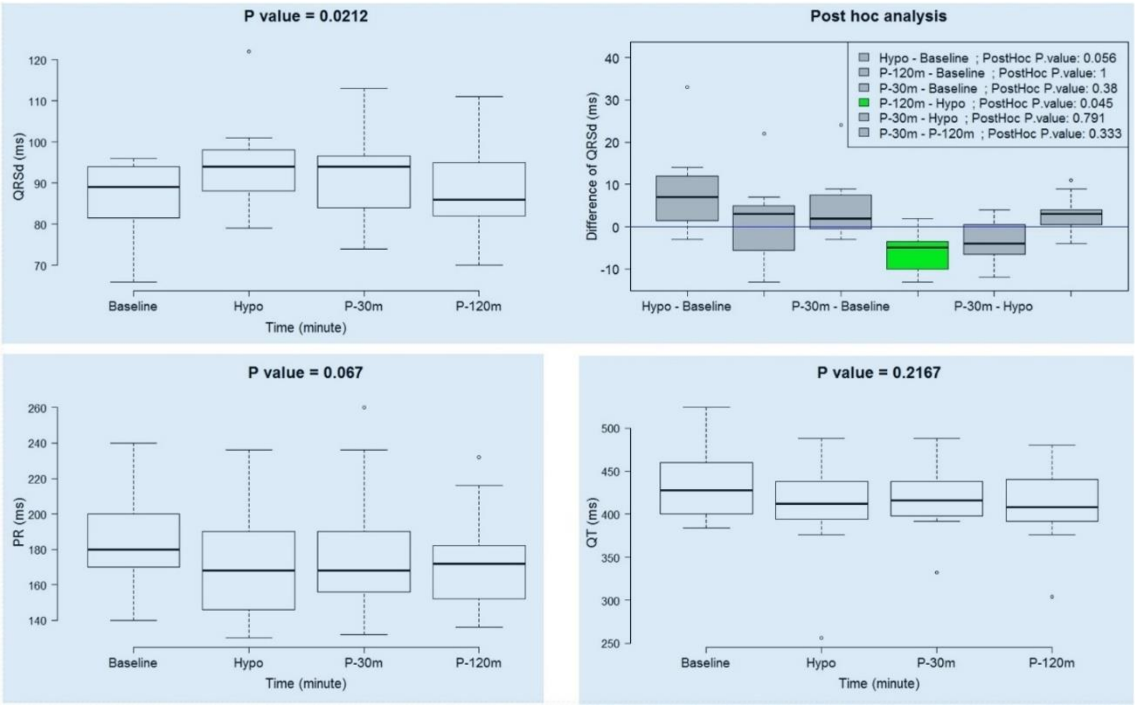

P-value was calculated by Friedman test. Hypo, hypoglycemia; P-30m, 30 minutes after hypoglycemia; P-120m, 120 minutes after hypoglycemia.
